# Hallucinations of Childhood

**Published:** 1872-10

**Authors:** 


*Hallucinations of Childhood.* The Annual Dissertation read before the Connecticut State Medical Society. By H. M. KNIGHT, M. D., of Lakeville.

This is a very interesting and instructive article on some of the Hallucinations of Childhood, and is from the pen of a physician whose long experience in the care of imbecile children entitle him to speak with authority. In conclusion, he gives the following by no means rare specimen of the effects of the system of pushing which is practiced in some of our schools.

"A frail, delicate girl at school had ten studies! A system of marking was rigidly enforced, 100 being the maximum of good recitations, or perfection in recitation. A monthly report was sent home. All institution and social influences were brought to bear to stimulate to perfection. This girl was obliged to send home one report, in which it was announced to the parents that she lacked 2-100ths of perfection in one or two studies. She accompanied it with a letter of regret and self-condemnation, and expressed her determination to send better returns in the future. Alas! before the next month, disease had claimed its legitmate victim, and that poor over-tasked brain was enjoying such a period of rest as only the delirium of fever affords.

"What unparalleled outrage or unmitigated humbug attends much of this so-called education of our youth!

"Our children need to receive 'that sound education which should consist in the literal educing of the faculties of the mind, of a counteracting agency to the instincts;—one which co-ordinates the faculties of the mind, which gives exercise to reason and judgment, at the same time that it represses without ignoring the instinctive part of our nature.' Precocity is an actual danger, and should not be fostered as a wonderful evidence of talent."

